# Supplementary material for: Active site specificity profiling datasets of matrix metalloproteinases (MMPs) 1, 2, 3, 7, 8, 9, 12, 13 and 14
Source: Data Brief. 2016 Feb 22;7:299–310. doi: 10.1016/j.dib.2016.02.036 (PMC4777984; doi:10.1016/j.dib.2016.02.036)
Supplement: Supplementary file 10 — Supplementary material [file mmc10.zip › WebPICS_hMMP7_G_1%/P2prime.html]

 

PICS results


|  |  |
| --- | --- |
| **P2prime\_H**  4 in 163 sites   2.5 %    effects > 10 perc. pnts.  (vice-versa in brackets)  P1\_Q: 40.8 (10.8) |  |
| **P2prime\_I**  16 in 163 sites   9.8 %    effects > 10 perc. pnts.  (vice-versa in brackets)  P2\_K: 10.2 (11.6)   P1\_N: 11.4 (15.2)   P1\_R: 20.2 (18.0)   P3prime\_G: 19.5 (16.5) |  |
| **P2prime\_L**  20 in 163 sites   12.3 %    effects > 10 perc. pnts.  (vice-versa in brackets)  P3\_A: 20.4 (12.7)   P2\_Q: 13.3 (24.1)   P1\_C: 12.5 (62.7)   P1\_P: 13.9 (27.7) |  |
| **P2prime\_Q**  11 in 163 sites   6.7 %    effects > 10 perc. pnts.  (vice-versa in brackets)  P2\_S: 19.3 (16.4)   P1\_Q: 18.1 (13.3)   P3prime\_V: 19.3 (16.4) |  |
| **P2prime\_R**  15 in 163 sites   9.2 %    effects > 10 perc. pnts.  (vice-versa in brackets)  P2\_K: 11.4 (12.2)   P2\_N: 12.0 (13.9)   P1\_G: 12.6 (15.8)   P1\_K: 16.3 (14.3)   P3prime\_Q: 13.3 (18.1) |  |
| **P2prime\_T**  16 in 163 sites   9.8 %    effects > 10 perc. pnts.  (vice-versa in brackets)  P3\_V: 16.5 (11.0)   P2\_N: 10.8 (13.3)   P1\_A: 14.0 (12.4)   P1\_G: 11.4 (15.2)   P3prime\_V: 10.8 (13.3) |  |
| **P2prime\_V**  23 in 163 sites   14.1 %    effects > 10 perc. pnts.  (vice-versa in brackets)  P3\_A: -15.3 (-11.0) |  |
